# The geometry as an eyed fish feels it in spontaneous and rewarded spatial reorientation tasks

**DOI:** 10.1038/s41598-020-64690-1

**Published:** 2020-05-15

**Authors:** Valeria Anna Sovrano, Greta Baratti, Davide Potrich, Cristiano Bertolucci

**Affiliations:** 1 Center for Mind/Brain Sciences, University of Trento, Rovereto, Italy; 2 Department of Psychology and Cognitive Science, University of Trento, Rovereto, Italy; 3 Department of Life Sciences and Biotechnology, University of Ferrara, Ferrara, Italy

**Keywords:** Spatial memory, Intelligence

## Abstract

Disoriented human beings and animals, the latter both sighted and blind, are able to use spatial geometric information (metric and sense properties) to guide their reorientation behaviour in a rectangular environment. Here we aimed to investigate reorientation spatial skills in three fish species (*Danio rerio*, *Xenotoca eiseni*, *Carassius auratus*) in an attempt to discover the possible involvement of extra-visual senses during geometric navigation. We observed the fish’s behaviour under different experimental procedures (spontaneous social cued task and rewarded exit task), providing them different temporal opportunities to experience the environmental shape (no experience, short and prolonged experience). Results showed that by using spontaneous social cued memory tasks, fishes were not able to take advantage of extra-visual senses to encode the spatial geometry, neither allowing them short time-periods of environmental exploration. Contrariwise, by using a reference memory procedure, during the rewarded exit tasks, thus providing a prolonged extra-visual experience, fishes solved the geometric task, showing also differences in terms of learning times among species.

## Introduction

Vertebrate and invertebrate species are able to reorient themselves in a geometrically characterized environment (usually rectangular), taking advantage of its “metric” – longer/shorter – and “sense” – left/right – properties (see reviews^1,2^). The typical task consisted in placing a human being or an animal in a rectangular room or arena, where a visible goal-object is located at a given corner. Then, the goal-object is removed, and the subject is taken out, passively disoriented through clockwise and counter-clockwise rotations, and reintroduced into the arena. Hence, the subject is requested to find the correct (reinforced) corner. In the pure geometric version of this task, the four walls of the arena are homogeneous (e.g., having the same colour and texture). The correct solution to this spatial problem involves searching in correspondence of the correct corner and its geometric equivalent diagonally located with respect to the goal. Since these two corners have the same metric and sense characteristics (e.g., a long wall on the right and a short wall on the left), they are indistinguishable.

Investigations in insects and other vertebrates, have raised the hypothesis that geometric and non-geometric encodings could be the result of processes based exclusively on visual information^3–8^.

For many years now, fishes have been an excellent model to study spatial reorientation in a geometrically characterized environment^9–20^. Intriguingly, blind cavefishes have also shown to be able to detect the environmental metric-plus-sense properties, but through extra-visual modalities, such as the lateral line and the sense of touch^21^.

The lateral line is a particular mechanosensory system that detects hydrodynamic stimuli, such as weak water motions and pressure gradients, allowing aquatic animals (cyclostomes, fish, and amphibians) to navigate and perceive the underwater world^22–30^. The main sensory component of the lateral line is the neuromast, a structure located on the skin (the superficial neuromast) or in fluid-filled canals beneath the scales (the canal neuromast). Superficial neuromasts are present in distinct lines or groups on the head, trunk and caudal fin, while lateral line canals shape a network on the head and single canals on the trunk^31,32^. The morphological patterns of the lateral line systems and the number of neuromasts differ among fish species and probably represent an adaptation to various hydrodynamic environments^23,31,32^.

The aim of our experiments was to investigate non-visual spatial reorientation skills in three different species of eyed fishes (the zebrafish *Danio rerio*, the redtail splitfin fish *Xenotoca eiseni*, the goldfish *Carassius auratus*), to determine whether eyed fish remain tied solely to their sight to build their own living environment and the spatial relationships within it; or if other sensory modalities (as the lateral line) could be involved in the construction of an environmental geometry’s representation for the purpose of spatial reorientation, contrary to the idea of an exclusive visual processing in these specific behavioural tasks.

We decided to consider these three species for our experiment, because they are very common aquarium fish, easy to maintain in laboratory in a cost-effective manner. Interestingly, these different fish species originated in three separate environments. The zebrafish and the goldfish are oviparous species native of the Asia continent and prefer slow-flowing waters of lakes, ponds and marshes. Differently, the redtail splitfin fish is a viviparous species originating in Mexico and thus inhabits shallow waters with little or no aquatic vegetation. Both zebrafish and goldfish have a large number of neuromasts and their lateral line anatomy is well known^23,31,32^, while the redtail splitfin fish is the prevalent animal model used in visual experiments on environmental geometry’s encoding^9–12,15,18,20^.

Our behavioural experiments have been conducted in a transparent rectangular arena, by providing three different opportunities to explore the experimental environment. In the present experiment we also used two behavioural procedures: a spontaneous social cued memory task (a sort of working memory procedure^15–17,20^) and a rewarded exit task (a reference memory procedure), similar to those used in other visual geometric tasks with humans and animals, sometimes offering doubts about their comparability^9–14,18–20^. Interesting was the comparison between these two experimental procedures in visual conditions, since recently divergent results have been shown in fish, when considering the use of local landmarks in non-geometric spatial reorientation tasks^20^.

In *Experiment 1*, during the social cued memory task, the experimental subject had the possibility to see a conspecific confined in a goal corner (into a glass jar); then after being covered, disoriented and the conspecific was removed, the fish was released into the empty tank and the spontaneous spatial reorientation was observed. Each fish was observed for five consecutive daily sessions in a transparent rectangular arena, without environmental exploration between the acclimation/observation-phase and the test-phase. Note that the behavioural observation of the spontaneous choices over several consecutive sessions was different with respect to the usual procedure validated on a single day by Lee *et al*.^15–17^, offering the opportunity to evaluate time as an additional variable. In *Experiment* 2, fishes were observed using a similar method as in Experiment 1, except the fact that this time they were allowed to explore the environment for a short time-period between the conspecific acclimation/observation-phase and the test-phase. In *Experiment 3*, fishes underwent a reference memory procedure in the same transparent rectangular arena used in *Experiment 1* and 2, but having four small transparent exit-corridors, embedded in correspondence of the four corners, thus allowing fishes to leave the arena. Fish were trained to only choose the two geometrical correct corridors that allowed them to exit the apparatus and arrive to a rewarded outer area, until reaching a learning criterion.

Due to the absence of extra-visual environmental explorations, we expected that fishes could not solve the spatial task in *Experiment 1*, thus constituting a control condition. On the other hand, we expected success in solving the geometric task by increasing the time available to explore the environment and moving from a spontaneous social cued task with short exploration times before each test-phase (*Experiment* 2) to a rewarded exit task with prolonged exploration times until reaching a learning criterion (*Experiment 3*), with a maximum success in this last experiment. Moreover, of great interest was the comparison between the rewarded exit task (*Experiment 3*) and the spontaneous social cued task (*Experiment 1* and 2) over the five daily sessions, a procedural variant that was introduced here for the first time. In that case, we expected a possible improvement over time in the spontaneous social cued task only in *Experiment* 2, when the environmental exploration of the spatial contingencies in relation to a specific goal was available.

## Results

### Experiment 1: Non-visual geometric task without experience

In *Experiment 1*, 6 zebrafish (*Danio rerio*), 5 redtail splitfin fish (*Xenotoca eiseni*) and 5 goldfish (*Carassius auratus*) were observed in the transparent rectangular apparatus that had one glass jar at each of the four corners, with a social conspecific as reward at the correct corner C (social cued memory task), in absence of any exploration before each experimental trial.

**Figure 1:**
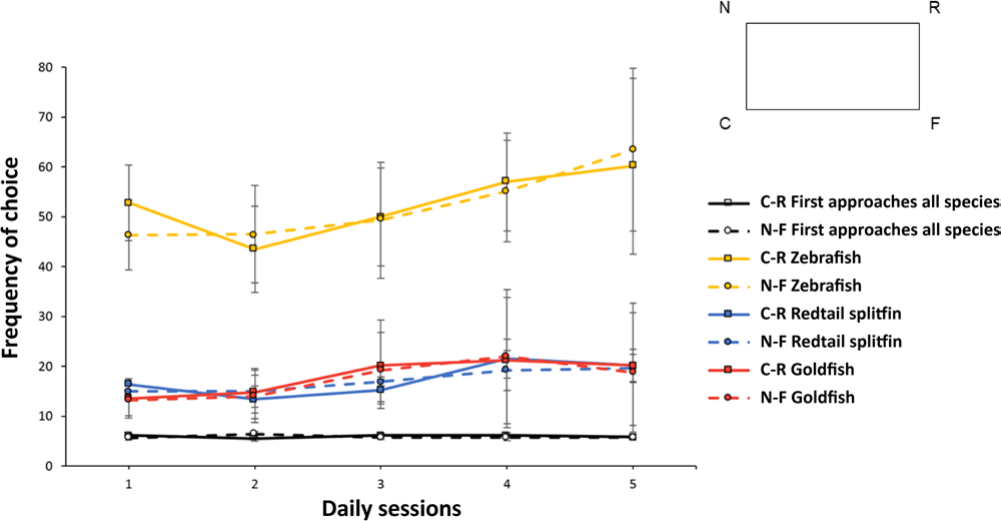
Results of Experiment 1. The graph shows the fishes frequency of choice (means with SEM), in terms of number of approaches at the geometrically correct diagonal (C + R) *vs*. the incorrect one (N + F), during five consecutive daily sessions in a transparent version of the social cued memory task, without exploration times. As shown in the schematic drawing, the geometrically correct corners are indicated with letter “C” (the correct corner) and letter “R” (the rotationally correct corner, on the same diagonal), while the incorrect corners are indicated with letter “N” (near the correct C) and letter “F” (far from the correct C). Black lines are referred to first approaches for all fishes together, yellow lines to approaches made by zebrafish, blue lines to approaches by redtail splitfin fish, red lines to approaches by goldfish. All fishes were not able to solve the social cued memory task – to encode the environmental shape in relation to a specific goal – in absence of extra-visual experience (i.e., through the lateral line and/or the sense of touch). Moreover, the number of zebrafish approaches is greater than that of the other two species considered.

Results of the *Experiment 1* are reported in Fig. 1.

The two correct corners are indicated with letter “C” (the correct corner) and letter “R” (the rotationally correct corner, on the same diagonal), while the incorrect corners are indicated with letter “N” (near the correct C) and letter “F” (far from the correct C).

The analysis of variance with Time (five daily sessions) and Geometry (diagonals C + R *vs*. N + F) as within-subjects factors, and Species (*D. rerio*, *X. eiseni*, *C. auratus*) as a between-subjects factor, applied on the first approaches only, did not reveal any significant main effect (Time: F(4,52) = 0.79, p = 0.53; Geometry: F(1,13) = 0.06, p = 0.81; Time x Geometry: F(4,52) = 0.37, p = 0.83; Species: F(2,13) = 1.14, p = 0.35; Time x Species: F(8,52) = 1.06, p = 0.4; Geometry x Species: F(2,13) = 0.11, p = 0.89; Time x Geometry x Species: F(8,52) = 0.58, p = 0.79).

The analysis of variance applied on the total approaches collected in 30 seconds, revealed instead a statistical main effect of Species (F(2,13) = 6.97, p = 0.009, ${\eta }_{p}^{2}$ = 0.52). All the other variables and their interactions were not significant: (Time: F(4,52) = 1.91, p = 0.12; Geometry: F(1,13) = 0.44, p = 0.52; Time x Geometry: F(4,52) = 1.67, p = 0.17; Time x Species: F(8,52) = 0.33, p = 0.95; Geometry x Species: F(2,13) = 0.03, p = 0.97; Time x Geometry x Species: F(8,52) = 1.51, p = 0.18). The difference among species was due to the high number of choices, for each trial over the five sessions, made by zebrafish (*D. rerio*), if compared to the other two species (*D. rerio:* mean ± SEM C-R = 263.5 ± 49.22 and N-F = 260.83 ± 49.66; *X. eiseni:* C-R = 86.8 ± 10.75 and N-F = 85.8 ± 9.66; *C. auratus:* C-R = 90 ± 40.67 and N-F = 87.2 ± 39.09). The swimming behaviour of zebrafish was very different compared to the one of other species studied here, because they have the tendency to move more rapidly respect to other freshwater species in novelty contexts^33,34^.

Results show that in the non-visual geometric *Experiment 1*, carried out in a transparent rectangular apparatus without any possibility to explore the environment before each test-trial, when the conspecific was still present (confined in the jar), fishes were not able to find the correct corner and its geometric equivalent, after the removal of the conspecific. This experimental condition has shown to be a good control condition because fishes could not experience, through extra-visual senses, the geometric arrangement of the experimental space in relation to a specific goal. Hence, results are consistent with such a control condition.

### Experiment 2: Non-visual geometric task with short experience

For this experiment naïve fishes, 6 zebrafish (*D. rerio*), 6 redtail splitfin fish (*X. eiseni*), and 7 goldfish (*C. auratus*) were observed in the same transparent rectangular apparatus having the four glass jars at the four corners, and with a conspecific placed in correspondence of the corner C (social cued memory task), as in *Experiment 1*. The difference here was the presence of short exploratory times (2 minutes) before each test-trial of each daily experimental session.

**Figure 2:**
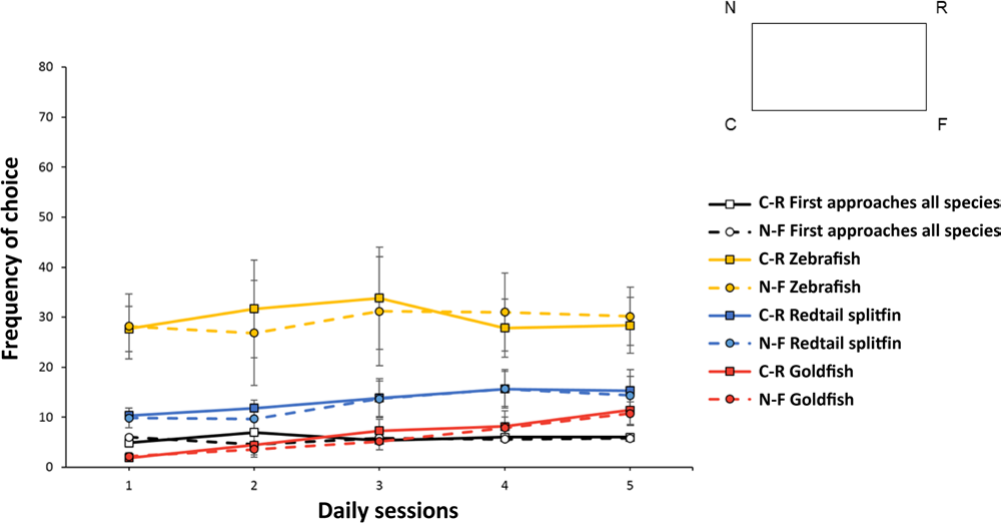
Results of Experiment 2. The graph shows the fishes frequency of choice (means with SEM), in terms of number of approaches at the geometrically correct diagonal (C + R) *vs*. the incorrect one (N + F), during five consecutive daily sessions in a transparent version of the social cued memory task, with a short exploration time before each test-trial. As shown in the schematic drawing, the geometrically correct corners are indicated with letter “C” (the correct corner) and letter “R” (the rotationally correct corner, on the same diagonal), while the incorrect corners are indicated with letter “N” (near the correct C) and letter “F” (far from the correct C). Black lines are referred to first approaches for all fishes together, yellow lines to overall approaches made by zebrafish, blue lines to approaches by redtail splitfin fish, red lines to approaches by goldfish. All fishes were not able to solve the social cued memory task – to encode the environmental shape in relation to a specific goal – also in case of short occurrences of extra-visual experience (i.e., through the lateral line and/or the sense of touch).

Results of the *Experiment* 2 are reported in Fig. 2.

The analysis of variance with Time (five daily sessions) and Geometry (diagonals C + R *vs*. N + F) as within-subjects factors, and Species (*D. rerio*, *X. eiseni*, *C. auratus*) as a between-subjects factor, applied on the first approaches only, revealed a marginally significant effect of the interaction Time x Geometry (F(4,64) = 2.52, p = 0.05, ${\eta }_{p}^{2}$ = 0.14). All the other variables and their interactions were not significant (Time: F(4,64) = 2.41, p = 0.06; Geometry F(1,16) = 0.39, p = 0.54; Species: F(2,16) = 0.87, p = 0.44; Time x Species: F(8,64) = 0.53, p = 0.83; Geometry x Species: F(2,16) = 0.42, p = 0.66; Time x Geometry x Species: F(8,64) = 0.59, p = 0.78).

Since the Species variable was not significant, the Wilcoxon test applied to the overall data (on the first approaches made by all fishes) could clarify the significant interaction Time x Geometry. The output of the Wilcoxon test, performed on each daily session separately, revealed an effect of Geometry in the second daily session (Z = −2.24, p = 0.03), but not in the other four sessions (first: (Z = −1.91, p = 0.06; third: Z = −0.08, p = 0.93; fourth: Z = −0.48, p = 0.63; fifth: Z = −0.41, p = 0.68).

On the other hand, as reported for *Experiment 1*, the analysis of variance applied on the total approaches collected in 30 seconds of test, revealed only a statistical main effect of Species (F(2,16) = 8.76, p = 0.003, ${\eta }_{p}^{2}$ = 0.52). All the other variables and their interactions were not significant: (Time: F(4,64) = 1.77, p = 0.14; Geometry: F(1,16) = 0.81, p = 0.38; Time x Geometry: F(4,64) = 2.26, p = 0.07; Time x Species: F(8,64) = 0.44, p = 0.89; Geometry x Species: F(2,16) = 0.03, p = 0.97; Time x Geometry x Species: F(8,64) = 1.05, p = 0.41). Also in this experiment, the difference among species was due to the differences of the total number of choices, in particular to the numerous choices made by zebrafish (*D. rerio:* mean ± SEM, C-R = 149.3 ± 32 and N-F = 147.3 ± 36.63; *X. eiseni:* C-R = 67 ± 12.36 and N-F = 63.2 ± 13.88; *C. auratus:* C-R = 33.14 ± 8.32 and N-F = 29.43 ± 6.09).

In the non-visual geometric *Experiment 2*, carried out in a transparent rectangular apparatus with short times available (2 minutes before each test-trial) to explore the environmental geometry when the conspecific was still present (confined in the jar), the results only of the first approaches seemed to show some effect of short explorations, even if this was limited to a single day. It is interesting to note that the day was the same (the second) in all the three species. However, such effect was not so meaningful to be maintained in the subsequent daily sessions nor when the results of the overall approaches were considered during the 30 seconds of behavioural observation. In other words, it seemed that fishes could not use extra-visual senses to extrapolate the geometric contingencies of the experimental rectangular environment in relation to a specific goal, when they had little time to explore that environment.

### Experiment 3: non-visual geometric task with prolonged experience

For this experiment naïve fishes, 8 zebrafish (*D. rerio*), 8 redtail splitfin fish (*X. eiseni*) and 7 goldfish (*C. auratus*) were observed in the modified version of the transparent rectangular apparatus, thus having the four corridors at the four corners, in presence of free prolonged environmental explorations during each trial, until reaching a learning criterion (reference memory procedure). The learning criterion was set at 70% (greater or equal) of choices on the correct diagonal (C1-C2) in at least two consecutive daily sessions.

**Figure 3:**
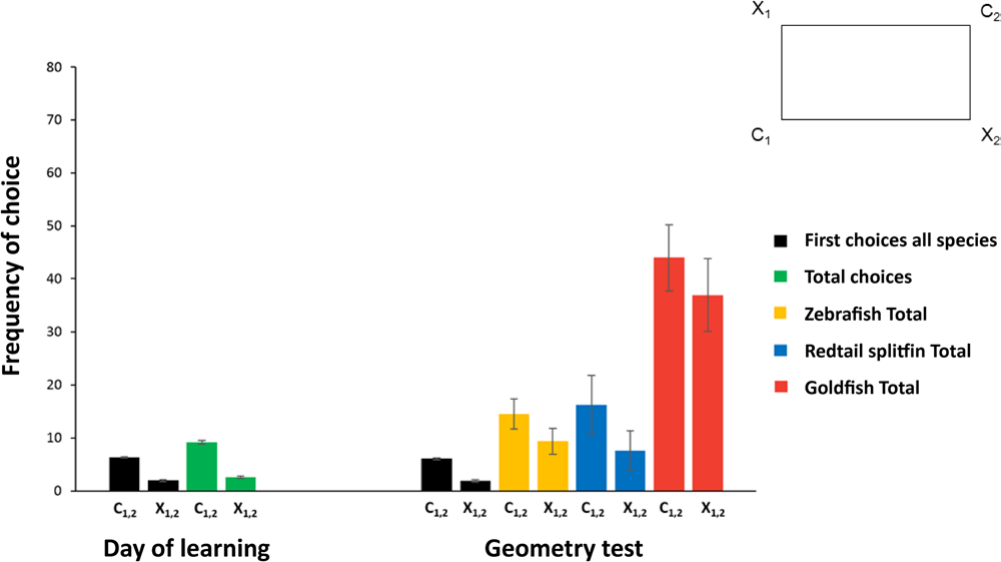
Results of Experiment 3 (day of learning and geometric test). Bar graphs show the fishes frequency of choice (means with SEM), in terms of number of attempts in correspondence of the corridors at the geometrically correct diagonal (C1 + C2) *vs*. the incorrect one (X1 + X2), during the Day of learning (when fishes reached the learning criterion) (on the left) and during the Geometric test (on the right), in a transparent rectangular apparatus. As shown in the schematic drawing, the geometrically correct corners are indicated with C1 and C2 (on the same diagonal, both open during the training-phase), while the incorrect corners with X1 (near the correct C1) and X2 (near the correct C2). Black bars are referred to first choices of all fishes together, green bars to total choices of all fishes together (where there was no difference among species), yellow bars to overall choices made by zebrafish, blue bars to choices by redtail splitfin fish, red bars to choices by goldfish. Fishes learned to use geometry of the transparent arena to reorient themselves: they were able to solve the non-visual rewarded exit task, achieving the learning criterion. The geometric test confirmed that the learning effectively occurred during training, although the number of attempts among species was different in extinction of response.

**Figure 4:**
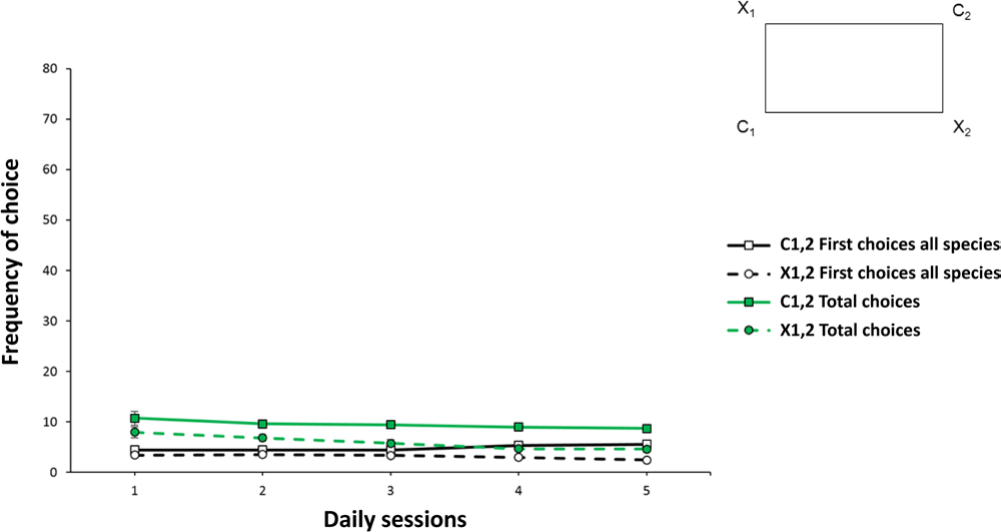
Results of Experiment 3 (training over first five daily sessions). The graph shows the frequency of choice (means with SEM), in terms of number of attempts in correspondence of the corridors at the geometrically correct diagonal (C1 + C2) *vs*. the incorrect one (X1 + X2), during five consecutive daily sessions under a reference memory procedure, in a non-visual rewarded exit task, with unlimited exploration times. Black lines are referred to first choices for all fishes together, while green lines to total choices for all fishes (there was not difference among species). The improvement of performance in the rewarded exit task began to become apparent from the first days of training with extra-visual experience (i.e., through the lateral line and the sense of touch) of the environmental shape.

Results of the *Experiment 3* are reported in Figs. 3 and 4.

The geometrically correct corners are indicated with C1 and C2 (on the same diagonal, both corridors were open during the training-phase), while the incorrect corners with X1 (near the correct C1) and X2 (near the correct C2), on the same incorrect diagonal (both corridors were closed during the training-phase).

The number of trials needed to reach the learning criterion was entered in a Univariate ANOVA with Species (*D. rerio, X. eiseni, C. auratus*) as a between-subjects factor. The ANOVA revealed a statistically significant difference among Species in reaching the learning criterion (F(2,20) = 29.34, p ≤ 0.0001, ${\eta }_{p}^{2}$ = 0.75; in fact, mean ± SEM, *D. rerio*: 28.6 ± 3.4; *X. eiseni*; 36.6 ± 4.8; *C. auratus*: 101.3 ± 11.9).

Results of the learning day (the single session where fishes completed the training) are reported in Fig. 3 (on the left).

The analysis of variance (ANOVA) with Geometry (diagonals C1 + C2 *vs*. X1 + X2) as a within-subjects factor and Species (*D. rerio*, *X. eiseni*, *C. auratus*) as a between-subjects factor, applied on the first choices only, revealed a significant main effect of Geometry (Diagonals C1 + C2 *vs*. X1 + X2: F(1,20) = 387,21, p ≤ 0.0001, ${\eta }_{p}^{2}$ = 0.95), while no other effect was significant (Species: (F(2,20) = 0.21, p = 0.81; Geometry x Species: (F(2,20) = 0.34, p = 0.72). The Wilcoxon test for paired data applied on the first choices made by all fishes did not reveal differences between the two correct corners C1 and C2 (Z = −1.11, p = 0.26) and between the two incorrect corners X1 and X2 (Z = −0.45, p = 0.72), while it emphasized the variable Geometry as effective (Z = −4.35, p ≤ 0.0001).

The same ANOVA applied on the total amount of attempts to find the correct corners confirmed the results obtained on the first choices: there was only a significant main effect of Geometry (Diagonals C1 + C2 *vs*. X1 + X2: F(1,20) = 563,52, p ≤ 0.0001, ${\eta }_{p}^{2}$ = 0.97), while no other effect was significant (Species: (F(2,20) = 1.62, p = 0.22; Geometry x Species: (F(2,20) = 3.1, p = 0.07). The two-tailed paired t-test applied on total choices made by all fishes did not reveal differences between the two correct corners C1 and C2 (t(22) = 0.83, p = 0.42) and between the two incorrect corners X1 and X2 (t(22) = − 0.12, p = 0.91).

When the training procedure was completed, fishes underwent a geometric test, in order to confirm the occurred learning in transparency conditions. Results of the geometric test are shown in Fig. 3 (on the right).

The analysis of variance (ANOVA) with Geometry (diagonals C1 + C2 *vs*. X1 + X2) as a within-subjects factor and Species (*D. rerio*, *X. eiseni*, *C. auratus*) as a between-subjects factor, applied on the first choices only, revealed a significant main effect of Geometry (Diagonals C1 + C2 *vs*. X1 + X2: F(1,20) = 101.75, p ≤ 0.0001, ${\eta }_{p}^{2}$ = 0.84), while no other effect was significant (Species: (F(2,19) = 0.21, p = 0.81; Geometry x Species: (F(2,19) = 0.86, p = 0.44). The Wilcoxon test for paired data applied on first choices made by all fishes did not reveal differences between the two correct corners C1 and C2 (Z = −0.75, p = 0.45) and between the two incorrect corners X1 and X2 (Z = −0.87, p = 0.39), while it emphasized the variable Geometry as effective (Z = −4.15, p ≤ 0.0001).

The ANOVA applied on the total amount of choices confirmed the significant main effect of Geometry (Diagonals C1-C2 *vs*. X1-X2: F(1,19) = 40.17, p ≤ 0.0001, ${\eta }_{p}^{2}$ = 0.68), but in this case, also the variable Species was significant (F(2,19) = 12, p ≤ 0.0001, ${\eta }_{p}^{2}$ = 0.56), while the interaction Geometry x Species was not (F(2,19) = 0.95, p = 0.4). The difference among species concerned the number of attempts at the geometric test, which was higher in goldfish (mean ± SEM, *D. rerio:* C1-C2 = 14.5 ± 2.83 and X1-X2 = 9.38 ± 2.46; *X. eiseni:* C1-C2 = 16.25 ± 5.61 and X1-X2 = 7.6 ± 3.7; *C. auratus:* C1-C2 = 44 ± 6.24 and X1-X2 = 37 ± 6.81.) The two-tailed paired t-test applied on total choices made by all fishes did not reveal differences between the two correct corners C1 and C2 (t(21) = 0.23, p = 0.82) and between the two incorrect corners X1 and X2 (t(21) = 2.03, p = 0.06).

Results provided by the geometric test confirmed that learning occurred during the training-phase, even if, because of the nature of this test under extinction procedure (in absence of reward), probably the first attempts alone could be considered as good predictors of learning consolidation during the previous training-phase.

Performance over time in the first 5 days of training is reported in Fig. 4.

When considering data for the two diagonals (C1 + C2 *vs*. X1 + X2) per fish in the summed first five training sessions, as done in the previous *Experiments 1* and *2*, the ANOVA with Time (five daily sessions) and Geometry (diagonals C1 + C2 *vs*. X1 + X2), as within-subjects factors, and Species (*D. rerio*, *X. eiseni*, *C. auratus*) as a between-subjects factor, applied on the first choices only, it revealed three statistically significant effects: Time (F(4,80) = 2.69, p = 0.037, ${\eta }_{p}^{2}$ = 0.12), Geometry (F(1,20) = 18.44, p ≤ 0.0001, ${\eta }_{p}^{2}$ = 0.48) and Time x Geometry (F(4,80) = 3.04, p = 0.022, ${\eta }_{p}^{2}$ = 0.13), while other variables or interactions were not significant (Species: F(2,20) = 0.06, p = 0.94; Time x Species: F(4,80) = 0.88, p = 0.53; Geometry x Species: F(2,20) = 3.12, p = 0.07; Time x Geometry x Species: F(8,80) = 1.78, p = 0.92).

The same ANOVA applied on the total attempts revealed three statistically significant effects: Time (F(4,80) = 7.19, p ≤ 0.0001, η^2^_p_ = 0.27), Geometry (F(1,20) = 24.86, p ≤ 0.0001, ${\eta }_{p}^{2}$ = 0.55) and Time x Species (F(8,80) = 3.94, p = 0.001, ${\eta }_{p}^{2}$ = 0.28), while no other variables or interactions were significant (Time x Geometry: F(4,80) = 0.74, p = 0.57; Species: F(2,20) = 2.25, p = 0.13; Geometry x Species: F(2,20) = 1.78, p = 0.19; Time x Geometry x Species: F(8,80) = 0.52, p = 0.84).

In the *Experiment 3*, in presence of prolonged environmental exploration in each trial, until reaching a learning criterion (reference memory procedure), in a transparent rectangular apparatus and after modifying the four corners in exit-corridors, results of the learning day showed that, without providing time limits to solve the spatial task, fishes learned the position of the reinforced diagonal on the basis of the geometry of the apparatus, detecting it through extra-visual senses. Although this occurred with different learning times depending on the species: e.g. goldfish needed more experimental trials to reach the learning criterion, thus confirming what was found in visual conditions by Vargas *et al*.^13^.

Results of the confirmation geometric test supported the hypothesis that learning occurred on geometric basis, even though there was a different behavioural tendency among species, in terms of attempts of choice (higher for goldfish). Interestingly, after a long adaptation to the environment during the training sessions, under a reference memory procedure (with the considerable increase of environmental exploration contingencies), zebrafish (*D. rerio*) reduced their motor activity, probably decreasing the emotionally response to novelty^33^. The same emotionally reduced behaviour was observable also in the visual version of the geometric test carried out over time^19^.

Analysing the performance trend in the first 5 days of training, as done in the two previous experiments, a progressive improvement in learning over time, that was shared among species, appeared.

## Discussion

It makes sense to assume that eyed fishes probably use sight as a priority sensory channel for building the spatial characterization of their living environment. Moreover, investigations in insects and vertebrates have raised the hypothesis that geometric and non-geometric spatial encodings could be the result of exclusively visual processes^3–8^. Contrary to this hypothesis, blind cavefishes were able to use extra-visual senses, such as the lateral line and/or the sense of touch with the pelvic fins, in order to learn how to solve the traditional geometric task^21^. If also eyed fishes would prove to be able to use their extra-visual senses to encode the environmental geometry, although their sensory preferential channel is the visual one, this would be a further element contrary to the involvement of a purely visual processing in the solution of geometric spatial reorientation tasks.

The crucial issue of our work was to investigate if eyed fishes, as well as blind cavefish^21^, were able to use their extra-visual senses to solve the same geometric task, disambiguating the two diagonals of a rectangular apparatus in environmental transparency conditions. As known, the two corners lying on the reinforced diagonal of a rectangular arena have the same geometric properties (“metric” – longer/shorter – and “sense” – left/right: for example, “the correct corners have a short wall on the left and a long wall on the right”).

For this purpose, two experimental procedures were applied to the behavioural observation of three fish species: a sort of working memory procedure (*Experiment 1* and *2*), already used with *X. eiseni* and *D. rerio*^15–17^, based on the spontaneous choices made by animals in a social cued task, and a reference memory procedure (*Experiment 3*) in a rewarded exit task, applying an operant conditioning with extended learning times, a technique well standardised even with *X. eiseni*, *D. rerio*, and *C. auratus* in similar geometric tasks^9–14,18–20^.

Differently from the standard social cued memory task used by Lee *et al*.^15–17^ that observed fishes on a single day, here we chose to extend the investigation to five consecutive days, in order to assess whether the repeated experience could play a role in this experimental procedure.

In *Experiment 1*, fishes were observed in the transparent rectangular apparatus with a conspecific as a social reward placed at the correct corner C (social cued memory task), in absence of exploratory times before each test-trial. When the conspecific was removed and the experimental animal released in the arena, the behavioural analysis at each corner showed that fishes randomly distributed their approaches among the four transparent jars at the corners, thus being unable to find both the correct corner and its geometric equivalent (on the same diagonal). The only effect we found, was the difference in the overall number of approaches performed by the three fish species considered. In fact, the total approaches were many more in zebrafish, which tended to show a typical motor pattern of high swim activity (i.e., erratic movements) in a novelty context^33–35^. This first experimental condition, where fishes could not experience the shape of the transparent rectangular environment in relation to a specific goal (social companion) through extra-visual senses, was a control condition for the subsequent experiments that were here performed. Moreover, it replicated results obtained on a single day of observation in similar studies engaging visual transparency conditions, as carried out by Lee and collaborators^16,17^.

In *Experiment 2*, fishes were observed in the same apparatus with the same procedure of *Experiment 1*, but this time they had the opportunity to explore the arena for a short time (2 minutes) before each test-trial, with the presence of the social companion at the correct corner. The behavioural analysis, applied on the first approaches only, seemed to show an effect of the short exploration, uniformly shared among all the species, even if limited to a single day (the second one). However, the effect was not maintained over time and completely absent when considering the total approaches. As in the previous experiment, the overall number of approaches was greater in zebrafish. In general, in this second experimental condition (social cued memory task with short exploration time), it seemed that fishes were not able use extra-visual senses to encode the geometric properties of the environment in relation to a specific goal, spatially characterized.

In *Experiment 3*, fishes were observed, under a reference memory procedure, in a transparent rectangular apparatus with four exit-corridors placed at the corners with a free prolonged exploration time available. Fishes were required to reach a learning criterion which was set at 70% (greater or equal) of choices at the correct corners C1 and C2 (in correspondence of the two corridors on the correct diagonal), which allowed them to exit the transparent apparatus and to swim in the surrounding area (where social rewards and food were provided after correct choices only). Results showed that, providing a long time to solve the spatial task, fishes learned the position of the geometrically correct corners, basing their searching behaviour on the shape of the transparent apparatus. In such visual transparency condition, there was a good chance that fishes detected metric-plus-sense properties through extra-visual senses. However, different species needed different time thresholds (in terms of number of trials) to learn and solve the geometric task: goldfish needed more than the double amount of time compared to zebrafish and redtail splitfin fish. A longer learning time in goldfish is strongly consistent with the known literature^13^, thus representing a particular species-specific trait in navigation by geometry. When the training procedure was concluded, fishes underwent a geometric test (in extinction of response), with the aim to confirm the occurred learning in visual transparency conditions. The results of this geometric test supported the behavioural data that fishes learned to encode the geometric shape of the experimental space during the training-phase, although different behavioural patterns among species emerged. These differences were measurable in terms of number of attempts at corners: indeed, in the geometric test, goldfish performed a greater number of choices with respect to zebrafish and redtail splitfin fish. Probably, under an extinction procedure (with all exit-corridors closed) and after a long training, the greater length of goldfish affected in some way the displacement times in the experimental area, favouring a huge number of attempts. Another possible explanation may regard the insensitivity to changes in conditions of reinforcement, as proposed by some authors^36,37^. They found that goldfish were not so responsive to aftereffect variations: for instance, fish showed no disruption of learning if shifted from large to small rewards. In other words, it is likely that a decrease in the amount of rewards (no rewards in our procedure) did not impair the performance because larger rewards (more than double of training trials needed to reach the criterion) had already produced stronger associative connections^38^. On the other hand, the reference memory procedure had markedly influenced the typical fishes’ behavioural pattern, in particular of zebrafish. It seemed that under an operant conditioning zebrafish had reduced the amount of their swimming activity in favour of better goal-oriented movements, probably as a habituation response to novelty and an emotionality reduction over time^33,34^. In fact, such a reduction of the swimming activity in zebrafish is also present in the visual version of this geometric task^19^. Moreover, analysing the time trend in the first five days of training, as done in the two previous experiments, there was a progressive improvement of learning over time, although in a different way depending on the species: as reported above, the learning performance was slower and less evident in goldfish, which needed a greater amount of trials to reach the learning criterion, as already observed by Vargas and collaborators^13^ in the visual version of the geometric task.

The overall results of these three experiments that were carried out under conditions of visual transparency in a rectangular apparatus, provide evidence that eyed fishes could encode the environmental geometry using extra-visual senses. However, fishes solved the task only when widely extended temporal contingencies in experiencing the position of a goal in relation to the geometry of the arena were allowed (reference memory procedure). Applying the traditional procedure of spontaneous choices in a social cued memory task^15–17^, even if repeated over time, it did not seem sufficient to guarantee the solution of the non-visual geometric task, through extra-visual modalities. In these conditions, one might also cautiously assume that the spontaneous use of non-visual cues may still be available, but it takes place only when vision cannot be used (i.e., in turbid water or in total darkness). It could actually be that processing of those cues is somewhat suppressed as long as visual signals predominate. On the other hand, although the experimental visual contingencies were different, a study conducted with children^39^ has shown that within a transparent rectangular room and by employing a working memory procedure of few trials in a single session, children used the geometric arrangement of transparent surfaces in relation to a specific goal-location at 5 to 7 years of age, but not before. In brief, it seems that such spatial skill could change during the human cognitive development.

In general, our results add evidence and confirm the great ecological value of environmental geometry encoding, as a useful tool for processing the structural environmental information^1,2^. In fact, such macroscopic cues (for instance, the presence of a mountain, a river, and their possible spatial relationships) remain constant over the span of an animal’s life, becoming crucial for survival.

Furthermore, results of the present study confirm previous findings collected with cavefish in similar tasks^14^, namely that the encoding of environmental geometry does not seem to be necessarily supported by processes computed on visual components^3–8^. Contrariwise, it is likely that non-visual geometric reorientation can be supported by extra-visual senses too, as the lateral line and/or the sense of touch with the pelvic fins. Both adult goldfish and zebrafish have a well-developed lateral line system composed of cranial and trunk canals as well as a proliferation of superficial neuromasts along all the length of the whole body that could be used to integrate spatial geometric information to navigate^31,40,41^. Furthermore, fins of fish play a role as tactile sensors by encoding pressures applied to the fin surface^42^. Functioning at different distances relative to the body, the lateral line and the fin mechanosensation could work in synergy to detect behaviourally relevant environmental characteristics. The specific role of one sense over the other one needs to be clarified, probably with pharmacological and/or electrophysiological techniques. This could be obtained by considering zebrafish as a useful organism model to investigate the role of different sensory modalities in spatial reorientation, given its widespread use in developmental biology and genetic.

Finally, our results also strongly emphasize that different behavioural procedures (working *versus* reference memory) can lead to different results in geometric spatial tasks investigating the use of extra-visual senses, if compared to visual geometric spatial tasks where the consistency of the geometric encoding remained under both procedures instead^9,10,15^. This is an important issue, that emerged here for the first time where we directly compared the two applied procedures. On the other hand, interesting recent results^20^ have shown that in the presence of visual local cues at the corners, without the influence of informative geometry through the use of squared environments, the ability to solve the reorientation spatial tasks, by finding the correct corner, remained better in rewarded exit tasks (reference memory) rather than in spontaneous cued memory tasks. When the visual geometric information was present, there was no difference in solution of the task under visual working and reference memory procedures, while in absence of visual geometric information (visual square apparatus), the performance in a rewarded task (reference memory) improved: just as it happened here but under transparent geometric conditions without visual coding.

Interestingly, it seems that in spatial reorientation, when visual or extra-visual channels processing the environmental purely geometric information are involved, the two behavioural procedures lead to different results.

## Methods

### Subjects and housing

The zebrafish *Danio rerio* and the goldfish *Carassius auratus* are two limnophilic freshwater cyprinid originating in Asia and are models widely used in behavioural and neurophysiological studies of lateral line functions. The zebrafish is native to the flood-plains of the Indian subcontinent, where it is found in slow-moving or standing water bodies, the edges of streams and ditches, particularly adjacent to rice-fields^43^. The goldfish may have originated from a natural mutation of an Asian carp in China approximately in the 960–1279 A.D.; it prefers slow-moving water of lakes, ponds and marshes^41^. The redtail splitfin fish *Xenotoca eiseni* is a viviparous cyprinodontiformes originating in north-central America. It occurs in spring-fed pools, streams, and lakes to depths of 1 meter^44^.

Here we used 20 mature males of zebrafish (4–5 cm in length), 19 mature males of redtail splitfin fish (2–4 cm in length) and 19 mature goldfish (4–6 cm in length). Due to the absence of sexual dimorphism, males and females of *C. auratus* were not distinguished. Fish were coming from breeding stocks in our laboratory: 6 *D. rerio*, 5 *X. eiseni*, and 5* C. auratus* participated only in *Experiment 1* (*“Non-visual geometric task without experience”*); 6 *D. rerio*, 6 *X. eiseni*, and *7 C. auratus* participated only in *Experiment* 2 (*“Non-visual geometric task with short experience”*); 8 *D. rerio*, 8 *X. eiseni*, and *7 C. auratus* participated only in *Experiment 3* (*“Non-visual geometric task with prolonged experience”*). In order to attract the experimental subject, conspecifics (one in *Experiments* 1 and 2, three in *Experiment* 3) were used as social stimuli. Fishes were maintained under a 10:14-h LD cycle and kept in glass-made home tanks (25 L capacity), enriched with gravel and cleaned with suitable filters (Aquarium Systems Duetto 100, Newa, I), in order to ensure a comfortable habitat. The water temperature was maintained at 26 °C with heaters (Newa Therm, Newa, I) and fishes were fed twice a day with dry food (GVG-Mix, Sera GmbH, D).

### Experiment 1: Non-visual geometric task without experience

#### Apparatus

**Figure 5:**
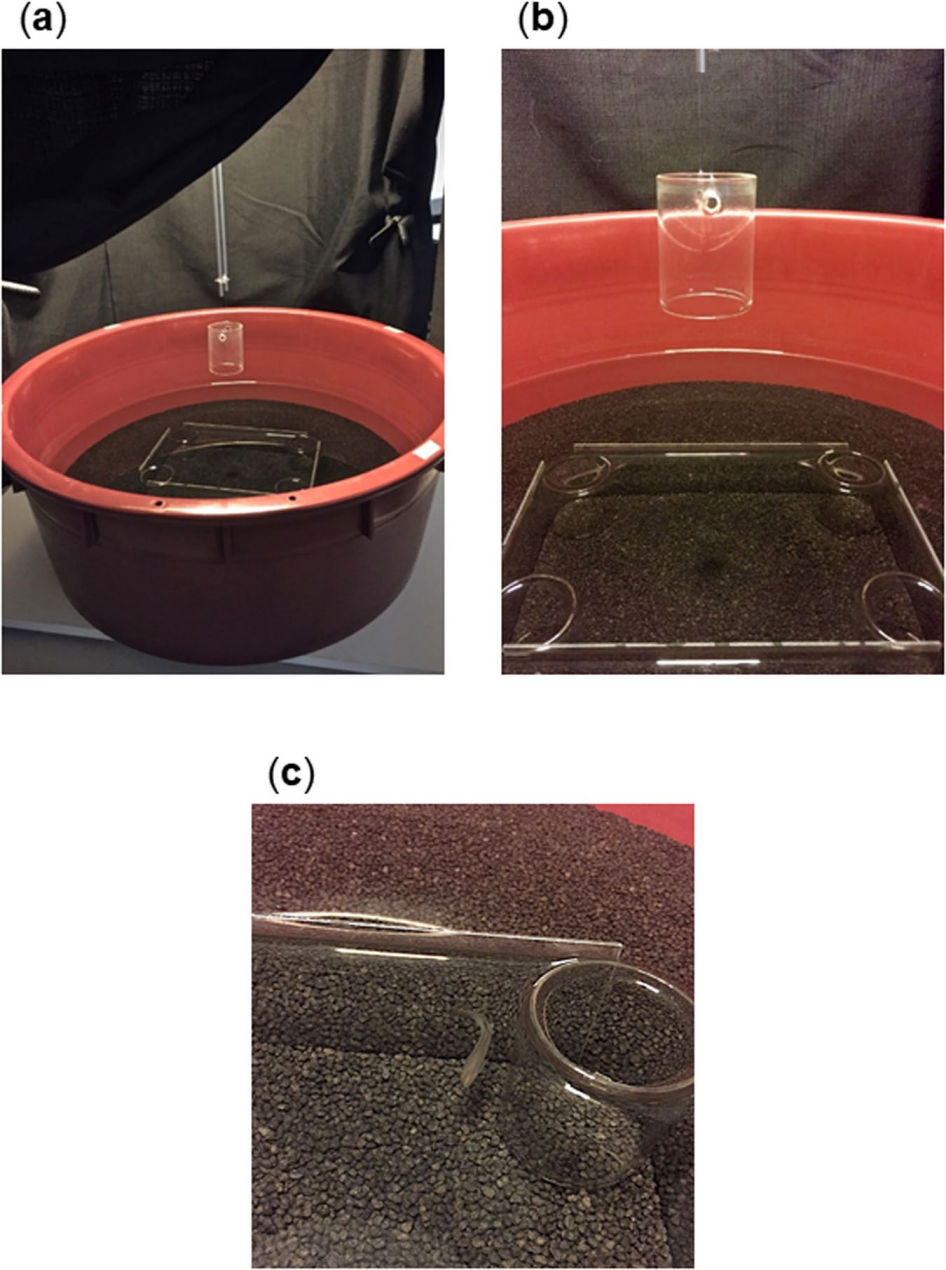
Transparent experimental apparatus for social cued memory tasks. Photographs of the transparent rectangular apparatus used for social cued memory tasks (*Experiment* 1 and *2*), completely submerged by water and inserted in a larger circular tank covered with black gravel on the floor; (**a**) the outside of the apparatus; (**b**) the transparent inner tank with four glass jars at corners and the glass cylinder that was used for housing the experimental fish during the observation period; (**c**) detail of a fish that is approaching the corner within 1 cm of the jar, therefore coded as an effective choice.

The apparatus was a transparent rectangular arena similar to the one used by Lee *et al*.^16,17^: specifically, it consisted of a rectangular enclosure (length: 30 cm; width: 20 cm; height: 8 cm) composed of four glass-made walls (length: short wall 16 cm, long wall 26 cm; height: 11 cm) (Fig. 5). In order to create a completely invisible environment, the four walls were not glued together but inserted in “single-track” supports fixed at the bottom of the experimental tank, then covered with dark gravel (depth: 5 cm). The transparent glass apparatus was inserted into a larger circular tank (diameter: 175 cm; height: 27 cm), creating an external surrounding region. At the four corners of the transparent arena four water-filled small glass jars were placed, (diameter: 6 cm; depth: 8 cm) one of them (placed at the correct corner C) could host the social attractor (Fig. 5). The apparatus, as well as the glass jars, was entirely inside the water for all its height (8 cm) but not submerged in order to create visual continuity in the absence of any light reflections.

In general, the two geometrically equivalent corners are those located on the same diagonal of the rectangular arena, having thus the same geometric – “metric” and “sense” – properties (e.g., a long wall on the right and a short wall on the left). The geometrically correct corners are usually indicated with the letter “C” (the correct corner) and the letter “R” (the rotationally correct corner, on the same diagonal); while the incorrect corners are indicated with letter “N” (near the correct C) and letter “F” (far from the correct C). For each species, one group of animals was reinforced on a diagonal, while another one was reinforced on the other diagonal.

In the centre of the apparatus a glass jar (diameter: 6 cm; height: 8 cm), which hosted the experimental fish, was located before the starting of each trial.

The experimental apparatus was placed in a darkened room, surrounded by a circular black curtain fixed to a metal structure and centrally lit from above (height: 100 cm) by a circular fluorescent white-light tube (24 W; Osram GmbH, D). The apparatus laid on a turntable, allowing the experimenter to rotate it (90° degrees, conventionally clockwise) at the end of each trial, in order to eliminate any potential extra-tank cue. The water temperature was maintained constant at 26 °C, while a filter (not present during the experiments) ensured a good water quality. A webcam (Life Cam Studio, Microsoft, I), fixed on top, recorded the fish behaviour.

#### Procedure

The procedure used was similar to the one used by Lee *et al*.^15–17^, with the main difference that in our task the behaviour of each subject has been observed for five consecutive sessions of twelve trials, rather than for a single day. In this way, the first session, allowed us to evaluate the performance in a pure spontaneous social cued memory task, while the subsequent ones were needed to clarify if the spatial behaviour could be eventually improved over time and influenced by repeated experience in the same experimental setting.

**Figure 6:**
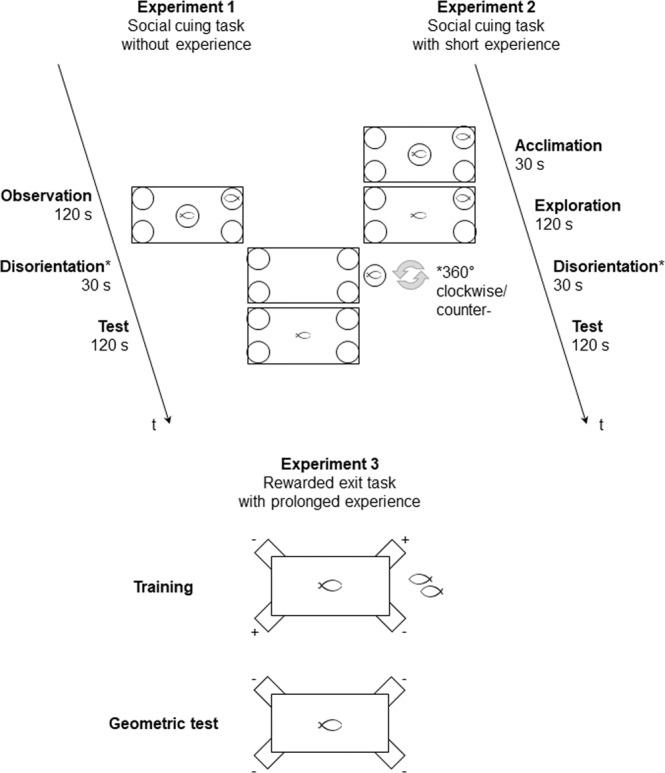
Experimental procedures. Schematic representation of the procedures used in Experiment 1, 2 and 3. The first and second experiment employed a social cued memory procedure (spontaneous approaches), while the third experiment employed a reference memory procedure (training over time). In details, Experiment 1 consisted of three periods: “observation” (120 seconds), where the fish was placed in a glass jar in the centre of the arena and could observe the social cue placed in a glass jar at a correct corner; “disorientation procedure” (30 seconds), where the fish was taken out from the arena and slowly rotate 360° clockwise/counter-clockwise, while the jar containing the social cue was replaced with a water-filled jar; “test” (120 seconds), where the fish was reinserted in the centre of the arena, free to approach the four jars placed at corners. Under such conditions, fish could not explore the arena to gain experience, but only observe the social cue. Experiment 2 was performed similarly but introducing two different periods before disorientation and test, that were “acclimation” (30 seconds), where the fish was placed in a glass jar in the centre of the arena and could accustom to the environment, and “exploration” (120 seconds), where the fish could swim in the arena and experience its shape in relation to the social goal for a short period through extra-visual modalities as, for instance, the lateral line and/or the sense of touch. Experiment 3 allowed fish to protractedly experience the rectangular environment and consisted of two phases: “training”, where the fish was trained to choose two geometrically equivalent corners (here indicated with “+”) to get a reward (food, conspecifics and prolonged inter-trial breaks), until reaching a learning criterion greater or equal to 70% of correct choices for two consecutive days of training; “geometric test”, in extinction of response (all corridors were closed; here indicated with “−”), with the aim to bear out that the learning had been achieved on geometric cues. Before each trial fish underwent the disorientation procedure (as done in *Experiment 1* and *2*), in order to reduce the use of compass and inertial information.

At the beginning of each trial, one conspecific, acting as a social attractor, was confined in one of the four jars located at the corners of the apparatus. Subsequently, the experimental fish was placed in the centre of the transparent arena, also confined in a glass jar, where it was allowed to observe the conspecific for two minutes (120 seconds), without being able to reach it (“observation” period) (Fig. 6). After the observation period, the jar containing the experimental subject was covered with an opaque blue plastic cylinder, gently carried outside the arena and slowly rotated, 360° both clockwise and counter-clockwise, in order to disorient the animal reducing the use of compass and inertial information (passive disorientation). In the meanwhile, the jar in the corner containing the conspecific was removed and replaced with an empty water-filled jar. The experimental fish was then gently transferred from the glass jar into a glass cylinder at the centre of the tank. At the upper end of the cylinder there was a transparent nylon wire that, through a stable and non-visible metal pulling mechanism, allowed a perfect vertical lifting upward of the cylinder. This procedure lasted thirty seconds. Finally, the cylinder was slowly lifted up, leaving the animal free to explore the apparatus for two minutes (“test” period), of which the first 30 seconds were later analysed^15–17^. At the end of each test trial the glass jar, containing the conspecific (the social cue), was placed in the correct corner C and the experimental fish was re-confined in the glass jar, in the centre of the arena, to start the next trial (Fig. 6). Before each trial, the whole apparatus was rotated 90 degrees clockwise with the aim to eliminate the possible influence of extra-tank cues.

Each trial was recorded and videos were subsequently coded by using a transparent grid where two “areas of choice” were drawn. In this way, it was possible to determine the portions of space within which an approach was considered as a preference: specifically, the approaches within 1 cm to the jar were considered as choices^15–17^. For each trial, the first approach (the first corner approached after the fish was released) and the overall approaches for corners C-R *versus* N-F in the 30 seconds of test were considered and analysed.

In Experiment 1, as well as in all the other experiments performed, an inter-observer reliability criterion^45^ was applied in the re-coding of a subset of 10% of different videos (*p* < 0.001, Pearson’s correlation between the ratio calculated on the original coding and on the *de novo* coding performed by an experimenter blind on the test condition of the fish).

### Experiment 2: Non-visual geometric task with short experience

#### Apparatus

The apparatus was the same as the one used in *Experiment 1* (Fig. 5).

#### Procedure

In *Experiment 2*, a slight procedural variant was applied, introducing a short exploration period: at the beginning of each trial, the animal was confined into the glass cylinder for 30 seconds of acclimation time (“acclimation” period) (Fig. 6). Subsequently, the cylinder was lifted, leaving the subject free to explore the arena (in presence of the conspecific in the jar at the correct corner) for two minutes (“exploration” period). After two minutes of exploration, the animal was delicately confined into the glass jar covered with the opaque blue plastic cylinder, passively disoriented, reintroduced into the glass cylinder in the centre of the tank and, thus, released for the two minutes of test (“test” period), after removing the jar with the conspecific and replacing it with the empty water-filled jar (Fig. 6).

Finally, the animal’s approaches for C-R versus N-F diagonals were coded exactly as in *Experiment 1* (first approaches, total approaches in 30 seconds of test during five consecutive daily sessions of 12 trials).

### Experiment 3: Non-visual geometric task with prolonged experience

#### Apparatus

In *Experiment 3*, the outer region surrounding the rectangular apparatus was crucial; when the fish swam out of the apparatus, the outer region was enriched with food, plastic plants, and three conspecifics (not subjected to experimental observations), providing a comfortable environment for the experimental fish and becoming an incentive to get out of the arena.

**Figure 7:**
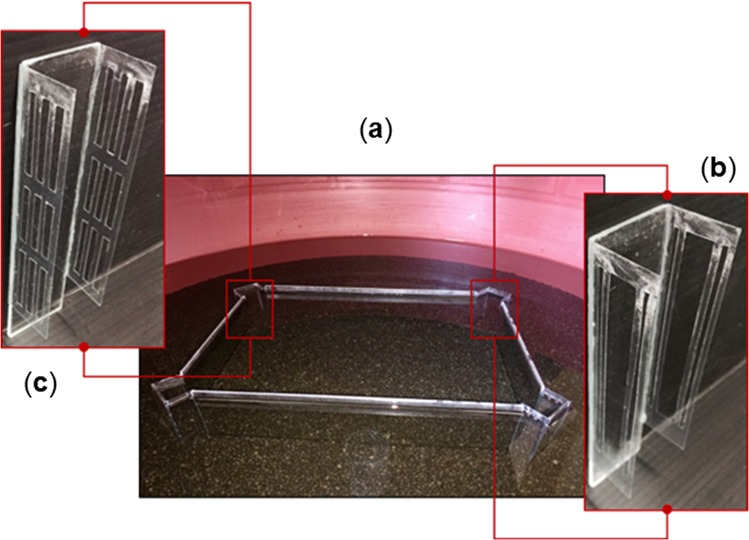
Transparent experimental apparatus for the rewarded exit task. Photographs of the transparent rectangular apparatus used under a reference memory procedure (*Experiment 3*), inside the water for all its height and inserted in a larger circular tank covered with black gravel on the floor; (**a**) the modified transparent apparatus with corridors at four corners. Around the experimental apparatus there was a comfortable environment for fishes (with conspecifics and food as rewards in case of correct choices), becoming an incentive to get out of the arena; (**b**) details of an open door; (**c**) details of a closed door.

At the four corners of the rectangular glass apparatus, instead of the glass jars, there were four small corridors, allowing the fish to leave the transparent arena and enter the external surrounding region. Each corridor was made of a rectangular transparent glass (3 × 11 cm), positioned towards the outside of the apparatus at 2.5 cm from the corner (3 cm considering *C. auratus* body length - BL) (Fig. 7a). On both longer sides of this rectangular glass, two rectangular transparent acetate sheets (2.5 × 9 cm; 3.5 × 11 cm considering *C. auratus* BL) were perpendicularly glued, with the aim to create a “C” shape corridor. The two acetate rectangles were carved, creating a vertical pattern with three series of rectangular fissures. The size of the fissures was different among the corridors, depending whether each corridor was assigned to the rewarded (i.e., the opened corridor where the fish can pass through it) or not rewarded corners (i.e., the closed corridor). More in details, the two open corridors had a large fissure in the middle (1 × 7.43 cm; 1.5 × 7.43 cm considering *C. auratus* BL) and two lateral fissures (0.2 × 7.43 cm), while the closed corridors had three superior and three inferior fissures (0.3 × 2.5; 0.355 × 2.5 cm considering *C. auratus* BL) and three central fissures (0.3 × 2 cm; 0.355 × 2 cm considering *C. auratus* BL; see details in Fig. 7b,c). Although the sizes of the fissures differed between the opened and the closed corridors, the overall fissures’ perimeter was balanced among corridors (47.4 cm; 48.4 cm considering *C. auratus* BL). This operation was needed in order to balance the potential effects of the water return generated by the swimming behaviour of fishes on their body sides, likely detectable by their lateral line in this task^21^. Thus, the open corridors were placed on the reinforced diagonal of the transparent rectangular apparatus, while the closed corridors were placed on the opposite, non-reinforced, diagonal. The terms “open” and “closed”, referred to the corridors, are useful to point out that only two out of four corridors allowed the fish to exit the experimental arena. The correct corners were indicated with C1 and C2, while the incorrect ones with X1 (near the correct C1) and X2 (near the correct C2). Different animals were reinforced with different diagonals. During the “geometric test”, following the training-phase and conducted with the aim to exclude any possible difference due to the amount of water filtered through the fissures and detected by the lateral line, the four corridors were modified. On both the long sides of the rectangular transparent glass (3 × 11 cm), two rectangles of transparent acetate without any fissures (2.5 × 9 cm; 3.5 × 9 cm considering *C. auratus*) were glued perpendicularly, as the corridors used for the training-phase. Such modified corridors did not allow fish to exit the arena and to be rewarded (extinction procedure).

#### Procedure

*Experiments 3* was divided in a “training-phase” and a “geometric test” (Fig. 6). The training-phase consisted of daily sessions of 8 trials until reaching the learning criterion (a number of correct choices greater than or equal to 70% for C1 + C2 – the two geometrically correct corners – in two consecutive daily sessions) and at least for a minimum of five consecutive daily sessions, in order to describe a learning curve for all animals, comparable with the performance achieved in *Experiment 1* and *Experiment 2*.

Before starting each experimental trial, the fish was gently transferred from the region surrounding the apparatus into a closed, opaque container (diameter: 13 cm; height: 7.5 cm), and passively disoriented (exactly as done in the two previous experiments). After the disorientation procedure, the fish was gently transferred in the glass cylinder in the centre of the inner transparent rectangular tank. After 30 seconds of acclimation, the cylinder was gently lifted up, leaving the fish free to move. In each trial, the number of choices for the four corridors was scored, until the fish was able to exit the tank, or for a maximum time of 10 minutes. A correction method was used^46^: after a wrong choice, the fish was allowed to change it, until it was able to exit the arena or until the maximum time for the trial elapsed. Inter-trials intervals, when the fish could remain in the external region, were 6 minutes (complete reinforcement time, with the administration of a small amount of food, the presence of three conspecifics and vegetation) if the correct corners only (C1 and C2) were identified; 2 minutes (reduced reinforcement time) if the correct corners were identified after two or more attempts. If the fish did not respond within the maximum time available (10 minutes), 5 minutes of pause-time in the outer region was given (without any reward) and the trial was considered null and repeated. In case of three consecutive null trials the daily experimental session ended. Multiple choices for the correct corridors could occur, for instance when fish explored them without actually getting out. An attempt to the corner was considered as an effective choice only if the animal exceeded half of the corridor, while the actual exit attempts were clearly visible in video-recordings, through characteristic tail and body movements.

After reaching the learning criterion, fishes underwent the geometric test, performed in two daily sessions of 4 trials each. At test, all the corridors were substituted with closed corridors without fissures (extinction procedure) (Fig. 6), in order to verify whether the learning had actually been achieved on the basis of geometric cues during the previous training-phase, rather than non-geometric ones (e.g., the different fissures’ pattern between opened and closed corridors). Each test trial lasted two minutes. If the animal did not make any choice in two minutes, the time available was prolonged, as long as it had not made at least one attempt, however time was not more extended than for a maximum of 10 minutes. If the fish did not choose any exit in this time, the trial was considered null and it was repeated. The inter-trial interval was 5 minutes, during which the fish was free to swim in the comfortable environment. To avoid a loss of motivation (since the corridors were not traversable during the extinction procedure), the test trials were interspersed with recall trials, carrying out the usual learning procedure with both the C1 and C2 corridors open.

The first choices and the total number of choices in correspondence of the four corridors per fish, in the training-phase (summing the first five sessions of 8 trials), during the learning day (the single session where fishes reached the learning criterion) and during the geometric test, were used as individual data, combining the approaches for the two diagonals (correct: C1 + C2; wrong: X1 + X2).

### Statistical analysis

The dependent variables measured In *Experiments 1* and *2*, for five daily sessions were: the first approaches (the first corner that fish approached after being released, in each trial) and the overall approaches for each diagonal (C + R *vs*. N + F) in 30 seconds of test; in the *Experiments 3*, the mean number of trials (with 95% CI) to reach the learning criterion greater or equal to 70% for C1 + C2; the first choices and the total number of choices for the two diagonals (C1 + C2 *vs*. X1 + X2) per fish, in the summed first five sessions of training, in the learning day (the single session where fishes reached the criterion) and in the single session of the geometric test.

In order to assess the homoscedasticity, the tests used were the Levene Test of equality of error variances and the Mauchly’s Sphericity test. The ANOVA was applied in order to compare the trend of geometric choices over time and among the three species. In the first choices, the non-parametric Wilcoxon test was applied in order to compare the corners on the same diagonals, while in the overall choices the Student’s t-test was applied with the same purpose. To estimate the effect sizes, we reported partial eta-squared (${\eta }_{p}^{2}$) as the index for ANOVA and 95% Confidence Intervals for Student’s t-test. Data were analysed with the IBM SPSS Statistics 20 software package.

### Ethical regulations

The present research was carried out in the Animal Cognition and Neuroscience Laboratory (A.C.N. Lab.) of the CIMeC (Centre for Mind/Brain Sciences) at the University of Trento (Italy). All husbandry and experimental procedures complied with European Legislation for the Protection of Animals used for Scientific Purposes (Directive 2010/63/EU). The University of Trento Ethics Committee for the Experiments on Living Organisms and the Italian Ministry of Health approved the experimental protocols (authorization number: 1111-2015-PR). The number of animals used in the experiments is closely consistent with the alternative method of “Reduction”, using only the minimum number of animals useful to draw statistically valid and powerful enough conclusions. All experiments were performed in accordance with relevant guidelines and regulations.

## Supplementary information

Supplementary Data.

## Data Availability

Data are available in a submitted supplementary Excel file.
